# Child Survival Crisis Due to Maternal Undernourishment During the COVID Era

**DOI:** 10.7759/cureus.31823

**Published:** 2022-11-23

**Authors:** Ashu Tyagi, Abhishek Joshi

**Affiliations:** 1 Preventive Medicine, Jawaharlal Nehru Medical College, Datta Meghe Institute of Medical Sciences, Wardha, IND; 2 Community Medicine, Jawaharlal Nehru Medical College, Datta Meghe Institute of Medical Sciences, Wardha, IND

**Keywords:** impact of malnutrition - mother and child nutrition, pregnancy and covid-19, malnutrition in pregnancy, maternal malnutrition, mother and child health, fetus, health, undernutrition, maternal, covid-19

## Abstract

The coronavirus disease 2019 (COVID-19) pandemic caused a global crisis, creating the most challenging times faced by any country. The pandemic created a situation that shocked the whole world. It led to a condition of fear, and the ones to take the major hit were the vulnerable groups: children, pregnant women, and the elderly, as well as those belonging to low socio-economic groups who lost their source of daily income. It increased the pressure on already burdened healthcare and information systems and led to a situation where the well-being of even children and pregnant women could not be maintained. COVID-19 increased the risk of undernutrition in children. Though children are observed to be less affected by the virus, they are the hidden victims of the pandemic in terms of falling prey to undernutrition. Child undernutrition can also be linked to maternal malnutrition, starting from the preconception period through the postpartum period. The situation arose due to the rapid steps of mitigation taken to tackle the pandemic, leading to decreased food security, healthcare, and education. Maternal undernutrition leads to complications for the mother during childbirth and has long-term effects on both. It can lead to low birth weight (LBW) babies, postpartum complications, chronic child undernourishment, and even increased maternal and child mortality and morbidity. Because of the pandemic's disruption of immunization facilities, it appears that even preventable childhood diseases will worsen in the coming years. In these post-COVID-19 times, it has become necessary to take measures to improve the overall health status of the population, with special regard to these vulnerable groups. Proper maternal and child health should be targeted at community levels by introducing interventions that prioritize antenatal and postnatal care, nutritional education, immunization of both mother and child, and proper health and sanitation practices. The purpose of this narrative review is to create awareness about the child survival crisis that may occur in the coming years due to undernutrition and the failure of immunization.

## Introduction and background

coronavirus disease 2019 (COVID-19), caused by SARS-CoV-2, is a contagious viral disease that has led to a pandemic and caused a catastrophe that not only led to the loss of many lives but also had an impact on the economies of all nations [1]. The developing countries, where low and middle socioeconomic groups form the majority of the population, were the ones most affected. This was caused by overburdened health care and health information systems. It led to the exhaustion of resources, disruption of work, and increased unemployment, which caused decreased food security due to decreased incomes and disrupted food supply chains, which led to a shift to less expensive sources of food. The most affected were women, due to loss of income and gender bias in all fields of work. There was decreased access to nutrition and food, even during the demanding preconception and pregnancy periods. Because of the reliance on spouses and families, the effects on mental health and family abuse have also increased. This condition was superseded by poor community health education and antenatal care services to support the mother. All these factors can lead to low-birth-weight infants, preterm births, chronic child malnutrition, and increased child morbidity and mortality. It also exerts long-term effects such as child maldevelopment, stunting, and decreased cognitive abilities, which will affect their work and contribute to the future economy [2,3]. The major factors that led to this situation are decreased food security and income, limited healthcare services and education, and an unhealthy environment [4].

This will turn out to be a major health consequence for children in the coming years. This can be attributed to maternal undernutrition during the natal period. Furthermore, the disruption of immunization programs will increase the risk of children contracting even preventable childhood diseases in the future. This can lead to a child survival crisis, risking the lives of many children, especially those who belong to lower-income groups. The purpose of this narrative review is to create awareness so that proper measures can be taken to assess the severity of the condition and improve it.

## Review

Normal nutritional requirements during pregnancy and lactation

Pregnancy is a physiological process during which the nutritional requirements of women change and affect the child's survival post-delivery and in the long term [2,5,6]. The nutrition requirements are divided based on macronutrients and micronutrients. Carbohydrates, protein, and fat are examples of macronutrients [7]. To meet the demands of pregnancy and the growing fetus, pregnant and lactating women must increase their caloric intake slightly. The resting metabolism, physical activity, and tissue growth of the fetus need to be met by adding adequate calories [8]. Protein is required to be taken in adequate amounts by pregnant women, as it is essential for the development of maternal tissue to support the fetus and for fetal growth. Increasing protein intake during the postpartum period is critical for the child's lactation. Fat is a macronutrient for which quality is more important than quantity when consuming it. The most studied of them is docosahexaenoic acid (DHA), which is important for fetal brain development. It is thus important for psychomotor development in the first month of life.

The micronutrients, though required in small quantities, are essential as well. One of the most commonly supplemented micronutrients is folate. It is essential for deoxyribonucleic acid (DNA) synthesis, and, thus, its deficiency can increase the risk of fetal anomalies such as neural tube defects like spina bifida. Vitamin A also needs to be supplemented by the mother, as it is required for normal bodily functions. It is important for vision, growth, metabolism, immune function, and the growth of maternal tissue to support the fetus. Vitamin B complexes are essential in pregnancy as they act as coenzymes in many metabolic pathways. Vitamin B12 functions with folate for the methylation of genetic material. Deficiency in these vitamins leads to maldevelopment in a fetus. The need for these vitamins increases during pregnancy to meet the increased energy and protein needs. Thiamine deficiency affects fetal brain development, while deficiencies in riboflavin and niacin are associated with preeclampsia, congenital heart defects, and low infant birth weight. Iron is one of the most important nutrients, as it is an integral part of hemoglobin and is required to maintain the oxygen demands of both the mother and fetus. It is deficient in most women and thus is the leading cause of complications during delivery. As a result, it is critical to start supplementing women before conception.

Vitamins C and E have an antioxidant effect and inhibit free radical formation. The complications associated with pregnancy are mostly related to oxidative stress; thus, they help prevent them. Vitamin C also plays a role in iron absorption and the laying down of ground matrices in extracellular tissues. Vitamin D is a hormone that plays a role in maintaining calcium metabolism within the body. During pregnancy, the fetus relies on vitamin D received from the mother. Thus, their deficiency leads to preterm births, preeclampsia, gestational diabetes mellitus (DM), or the birth of infants who have LBW and neonatal rickets. Calcium is essential in the process of bone mineralization and is a key intracellular component for maintaining cell membranes. It is also involved in several biological processes. Some recommend supplementation to preserve maternal calcium balance and bone density and support fetal development. Low maternal calcium intake can contribute to osteopenia, paraesthesia, muscle cramps, tetanus, tremors in the mother, delayed growth, LBW, and poor fetal mineralization. Idoine intake by the mother needs to increase as it is essential for the proper growth and development of the fetus, and its deficiency can lead to intellectual impairment and stunting in the child. Zinc is an essential mineral in various catalytic processes of metabolism, cellular development, and antioxidant effects. Zinc deficiency in pregnancy is known to cause poor immunity, prolonged labor, preterm and post-term births, and LBW. [9,10].

Weight gain in pregnancy is considered a good sign of development. A normal pregnant woman is supposed to gain between 5 and 9 kg because of the development of the fetus, placenta, amniotic fluid, and changes in maternal tissue. This is known as gestational weight gain [11]. Women with poor weight gain during pregnancy are at risk of preterm birth, while excess weight gain has been associated with gestational diabetes, postpartum hemorrhage, and obesity [12].

Maternal undernourishment status in the pre-COVID-19 era

Maternal malnutrition is one of the causes of the rampant child survival crisis across the globe, in both developing and developed countries. It is associated with poverty, food insecurity, poor sanitation, and an infectious disease burden in poor socioeconomic strata. Undernourished women fail to improve their nutritional status and fail to gain weight. Thus, persistent maternal undernutrition contributes to poor birth outcomes and childhood malnutrition in the long term. All malnourished children are classified as having underweight, stunting, wasting, or protein energy malnutrition. Micronutrient deficiency is another known cause of high-risk pregnancies. Among these, the most important are iron, vitamin A, iodine, and zinc deficiencies [13].

Impact of COVID-19 on nutrition

The pandemic raised a situation of concern for the whole community, and the major factors that led to this were socioeconomic deficits. The most affected were those depending on their daily source of income. All of this occurred due to the rapid interventions made by governments to tackle the pandemic. The major factors that affected nutrition are mentioned below.

Loss of Income of Low Socioeconomic Groups

The COVID-19 pandemic has created a situation that has not only caused loss of life but has also led to an economic crisis across the globe [14,15]. The developing countries, in which middle and lower socioeconomic strata groups form the majority of the population, are the most affected. This can be attributed to the disruption of essential health care and poor health information systems [16]. Long lockdown periods led to the disruption of supply chains, price hikes, and a decline in the overall amount of food consumed. People lost their source of income as a result of which they shifted to less expensive sources of calories such as cereals, oil, and processed foods and reduced their consumption of dairy and animal products. These poor-quality diets led to an increased risk of undernutrition and micronutrient deficiencies. Social protection programs such as cash and food transfers and school meals were disrupted by the pandemic. This pandemic has shown that all countries fall short of maintaining a certain level of living standards [14]. The ones to take the major hit are the dependable groups or the vulnerable groups, which include children, pregnant women, lactating women, and old people, due to various social and health inequities.

Increased Food Insecurity

When describing the after-effects of the pandemic, "food security" is a term that is most inherently being used to describe access to sufficient, safe, and nutritious food that meets the dietary needs of all people at all times to live a healthy life. The pandemic created a situation in which vulnerable groups' income and food security were severely impacted. The decreased food security has shown a negative effect on caregivers supporting the household. As a result, their nutrition and ability to feed their families suffered. It has also been associated with mental issues leading to violence with a partner and inadequate feeding of milk to infants. Poor food security causes an increased risk of chronic undernutrition and infectious diseases in children, anemia in mothers, and the development of non-communicable diseases like type 2 diabetes [17]. Massive poverty increases and reductions in cross-border trade, labor migration, and employment have resulted in major food system disruptions [18]. Most developing countries depend on farm workers and migrant laborers to plant and harvest their crops. The disruption of the movement of farm workers across countries led to a shortage in the availability of staple foods and fresh produce and increased consumption of ultra-processed foods. As a result of these restrictions, many lost their income sources and had no social protection to properly support them during the crisis [18]. The disruption of maternal, newborn, and child health services (MNCH) is also a major concern in developing countries. This could be due to the fear instilled in seeking health care but also to restricted transportation during lockdowns and the diversion of the healthcare system towards other essential activities to tackle the pandemic [19,20]. Maternal malnutrition is thought to be caused by food insecurity in developing countries, which resulted in the loss of an income source for daily wage workers working in both agricultural and non-agricultural fields. Further, this was superseded by price hikes for all goods. Due to restricted movements and disrupted supply chains, people resorted to cheaper and more accessible food sources, which have low nutritional value and more ill effects on health [15]. The pandemic has led to a global emergency by producing a state of economic despair, with a large proportion of the population falling below the poverty line. The ones most affected were women due to gender bias in all lines of work. This was exacerbated by limited access to mobility and services [21]. 

Overburdened Healthcare System

**Figure 1:**
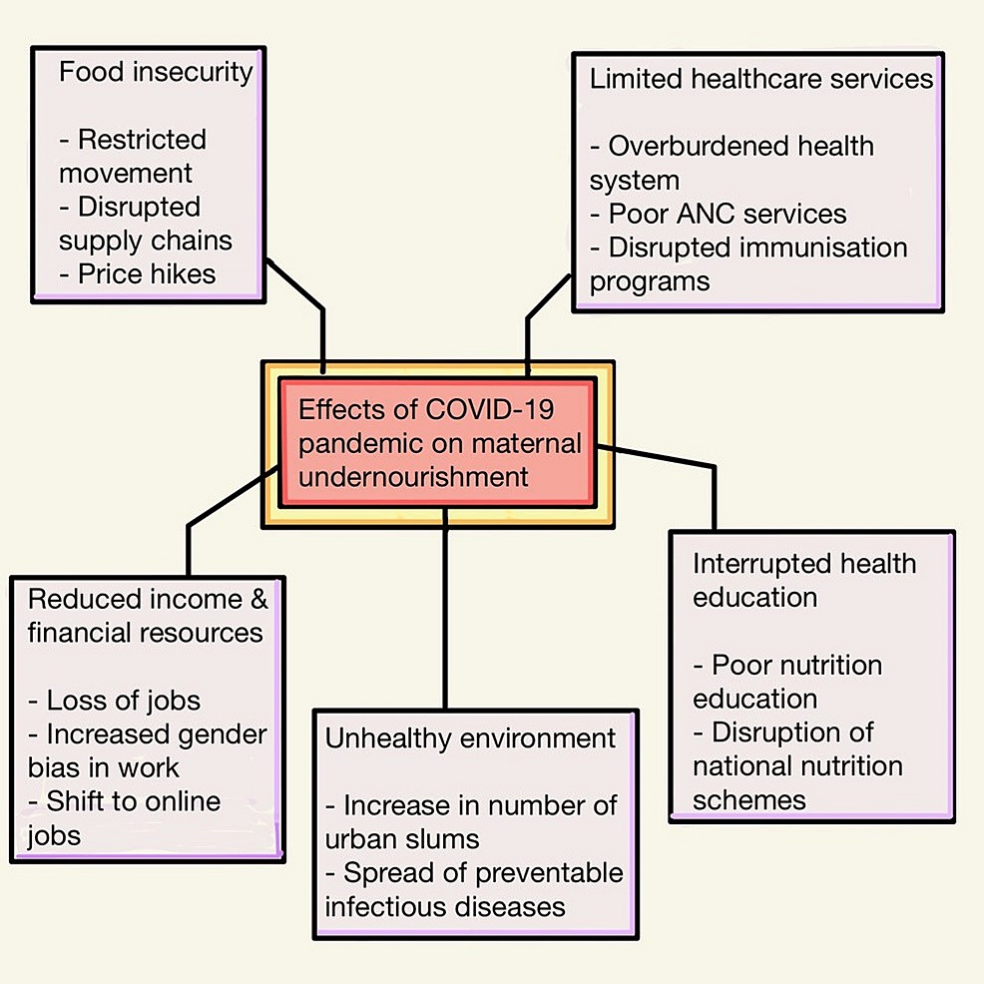
The impact of COVID-19 on maternal malnutrition Figure created by the author.

The health system was overburdened, leading to a shift from primary maternal and child care to dealing with COVID. Thus, with the current state of matters, child and maternal undernutrition and poor health services might prove to be bigger crises than COVID. Disruptions to routine and necessary maternal care and nutrition can lead to adverse outcomes, including preterm birth, low birth weight, and postpartum complications [22]. The disruption of educational services is another sector that took a major hit. Women and girls, who suffer a higher rate of school dropouts and illiteracy, are bound to suffer more as a result of the lack of necessary health education regarding nutrient education to improve maternal and child nutrition. Another setback is the disruption of school nutrition programs, which could deal with early undernutrition in poverty groups [23]. Funds for community health programs and the construction of safe, sanitary, and healthy households have been neglected as a result of the shift in priorities. This could lead to a worsening of the status of urban slums and poor households in rural areas. This, in turn, could also lead to the spread of infectious diseases in these vulnerable communities [24]. Figure 1 summarizes the impact of COVID-19 on maternal and child nutrition.

Long-term effects on child health

Maternal undernutrition is a concerning health problem in most developing countries. A mother's nutrition before conception, during conception, and after delivery affects a child's development in many ways. It not only increases the risk of maternal and child mortality but also has long-term effects on the child. One of the most important effects is stunting [25]. Poor nutrition impacts the brain's cognitive functions and causes long-term consequences like diminished learning capacity in both childhood and adulthood, causing reduced earnings and increased risks of diseases [26]. Maternal undernutrition is associated with higher risks of wasting, stunting, and being underweight in children. Stunting that occurs in the early years of life is largely irreversible and contributes to an intergenerational cycle of poor growth and development [27]. The probable mechanism for these long-term effects can be attributed to inadequate growth and tissue remodeling during critical periods of development due to inadequate substrates. Fetal growth occurs sequentially, and inadequate nutrition during any phase can cause maldevelopment [28]. This is explained by changes at the genetic level and subsequent epigenetic changes. These epigenetic changes occur during the formation of the embryo and are sensitive to the nutrition provided to the fetus by the mother. These abnormalities are thus preventable through proper maternal nutrition [29].

Interventions

All the countries in the world are trying to evolve with the pandemic and find ways to bring their lives back on track. The following are the ways in which this can be tackled [30-32]. Food insecurity interventions must be taken. Food security challenges vary by country and may be more severe in developing and underdeveloped countries. But the measures taken may overlap, as the ones that need to be targeted are the low socio-economic classes in all the countries. These may include an increase in investment in the agricultural sector and a cut in the government's expenses on other developmental programs, with the main focus on assisting the lower socioeconomic groups. Some long-term and short-term solutions for balancing supply- and demand-side challenges for nutrition protection could be considered [33].

Social protection programs that prioritize vulnerable groups to provide them with economic security must be launched. These measures can be viewed as social programs and tax breaks. Social protection programs are the most effective way to tackle poverty, vulnerability, and social exclusion. This, along with a well-developed health and data information system, can be more effective in ensuring the distribution of resources and the success of these programs. These health programs must aim to meet the healthcare needs of the masses so that they can be accessible to these people at all times, even in the most remote areas. This can be achieved by mobilizing community health workers to deliver the vaccine, nutritional supplementation, health education, and antenatal care. The number of both medics and paramedics should be increased so that the healthcare facilities can reach everyone and prioritize maternal and child health [34].

The closure of education systems must be compensated for with an increase in the education of the masses at the community level by community health workers. Proper nutritional counseling and healthy sanitary practices should be provided to the less educated. Special health education must be given to pregnant women regarding proper nutrition and antenatal and postnatal care. Safe and healthy household and community environments must be provided for. Another aspect to consider is infrastructure support and access to safe water and sanitation services (for example, through the construction of wells, community pipes, and latrines).

## Conclusions

The COVID-19 pandemic turned out to be an unprecedented crisis that shook the whole world. However, it taught all countries a lesson and highlighted the gray areas where they lacked. Long lockdowns and disruptions in food and primary healthcare systems have led to a global crisis. This is because maternal and child care have been neglected with the shift to dealing with the disease. This is all because of the overburdened healthcare system and exhausted resources. Maternal undernutrition due to decreased food security can lead to complications for the mother during childbirth and may have long-term effects on both mother and child. It can lead to low birth weight babies, postpartum complications, chronic child undernourishment, and even increased maternal and child mortality and morbidity. In this post-COVID-19 period, it is necessary to learn and come up with ways to live with the disease while maintaining a normal flow of life. This can only be achieved when the whole population is taken into consideration, with special provisions for vulnerable groups. Ensuring maternal and child health should be given primary importance, as they are the future of any country. This can only be achieved at the community level by introducing interventions that prioritize antenatal and postnatal care, nutritional education, immunization of both mother and child, and proper health and sanitation practices. Social programs and new strategies must be initiated and reformed to meet the needs of the hour. For the effective execution of these strategies, they need money, political will, commitment, and international unity to battle the pandemic and its after-effects by standing together and giving support.
